# Nanoparticles in the environment: where do we come from, where do we go to?

**DOI:** 10.1186/s12302-018-0132-6

**Published:** 2018-02-08

**Authors:** Mirco Bundschuh, Juliane Filser, Simon Lüderwald, Moira S. McKee, George Metreveli, Gabriele E. Schaumann, Ralf Schulz, Stephan Wagner

**Affiliations:** 1 Functional Aquatic Ecotoxicology, Institute for Environmental Sciences, University of Koblenz-Landau, Fortstrasse 7, 76829 Landau, Germany; 2 Department of Aquatic Sciences and Assessment, Swedish University of Agricultural Sciences, Lennart Hjelms väg 9, 75007 Uppsala, Sweden; 3 FB 02, UFT Center for Environmental Research and Sustainable Technology, University of Bremen, Leobener Str. 6, 28359 Bremen, Germany; 4 Ecotoxicology and Environment, Institute for Environmental Sciences, University of Koblenz-Landau, Fortstrasse 7, 76829 Landau, Germany; 5 Environmental and Soil Chemistry, Institute for Environmental Sciences, University of Koblenz-Landau, Fortstrasse 7, 76829 Landau, Germany; 6 Department of Analytical Chemistry, Helmholtz Centre for Environmental Research-UfZ, Permoserstrasse 15, 04318 Leipzig, Germany

**Keywords:** Nanomaterials, Co-contaminants, Environmental parameters, Review, Fate

## Abstract

Nanoparticles serve various industrial and domestic purposes which is reflected in their steadily increasing production volume. This economic success comes along with their presence in the environment and the risk of potentially adverse effects in natural systems. Over the last decade, substantial progress regarding the understanding of sources, fate, and effects of nanoparticles has been made. Predictions of environmental concentrations based on modelling approaches could recently be confirmed by measured concentrations in the field. Nonetheless, analytical techniques are, as covered elsewhere, still under development to more efficiently and reliably characterize and quantify nanoparticles, as well as to detect them in complex environmental matrixes. Simultaneously, the effects of nanoparticles on aquatic and terrestrial systems have received increasing attention. While the debate on the relevance of nanoparticle-released metal ions for their toxicity is still ongoing, it is a re-occurring phenomenon that inert nanoparticles are able to interact with biota through physical pathways such as biological surface coating. This among others interferes with the growth and behaviour of exposed organisms. Moreover, co-occurring contaminants interact with nanoparticles. There is multiple evidence suggesting nanoparticles as a sink for organic and inorganic co-contaminants. On the other hand, in the presence of nanoparticles, repeatedly an elevated effect on the test species induced by the co-contaminants has been reported. In this paper, we highlight recent achievements in the field of nano-ecotoxicology in both aquatic and terrestrial systems but also refer to substantial gaps that require further attention in the future.

## Introduction

Nano-based technology has made enormous progress over the last decades, which is underpinned by a 25-fold increase between 2005 and 2010 in the numbers of products that either contain or require nanoparticles (NP) for their production [1]. This development is likely facilitated by their unique general properties (in particular particle size, surface area, surface reactivity, charge, and shape) relative to their bulk or dissolved counterparts. This enables a broad range of possible applications, including cosmetic, pharmaceutical, and medical utilization [2, 3]. Engineered NP consist of carbon-based and inorganic forms, partly with functionalized surfaces [4].

Along with their unique general properties, the high diversity of NPs’ elemental and structural composition has, however, challenged environmental scientists in multiple ways, ranging from NP characterization and fate in complex matrixes [e.g. 5] to individual and combined NP effects in aquatic and terrestrial (eco)systems [e.g. 6]. The crystalline composition of titanium dioxide NP (TiO_2_ NP), for instance, influences their toxicity for the water flea *Daphnia magna* when based on the mass concentration—a pattern independent of the initial particle size [7]. Such observations suggest that other or additional dose measures, such as particle number, surface area, or body burden, are needed to adequately reflect the exposure situation [8]. In fact, NPs’ surface area explained a large share of variability in the resulting toxicity among crystalline structures [7].

In this paper, we give a brief overview of recent scientific advances enhancing the understanding of the (i) sources and (ii) fate of NP, (iii) the effects of NP in simplified studies, and (iv) how NP interact with biota in a more complex environment. We consider both aquatic and terrestrial systems but mainly focus on metal-based NP as carbon-based NP are covered elsewhere [9, 10]. We will, however, not specifically cover the methodological developments in the context of NP quantification and properties as this has also been covered elsewhere (see for instance, [11–13]). On this basis, we develop recommendations for future research directions in nano-ecotoxicology.

## Pathways of nanoparticles into natural ecosystems

It has been anticipated that the increasing application of NP both quantitatively but also in terms of product diversity will lead to a diversification in emission sources into the environment [4]. Key products containing NP are coatings, paints and pigments, catalytic additives, and cosmetics [14]. This chapter will discuss NP emissions from such products, whereas the release process is beyond our scope.

NP can enter the environment a long their life cycle and three emission scenarios are generally considered: (i) release during production of raw material and nano-enabled products; (ii) release during use; and (iii) release after disposal of NP-containing products (waste handling) [15–17]. NP emissions can be either directly to the environment or indirectly via a technical system such as wastewater treatment plants (WWTPs) or landfills. Indirect emissions are likely occurring either via the effluent of WWTPs, application of biosolids to soil, or leachates from landfills. It has also been pointed out that NP fate in technical systems such as WWTPs determines whether bare, coated, chemically or physically transformed particles are released, and via which pathway (as effluent or biosolid) [18–21].

So far, emission and environmental concentration levels have been estimated using material flow models following the NP life cycle [22]. Calculation models assume that produced NP will be released either to waste streams or directly to environmental compartments, and more realistic approaches account for the delayed release during use due to in-use NP stocks. NP emissions are also controlled by (i) ageing or weathering [19, 23–26], (ii) the fate of the NP during use [20, 27, 28], and (iii) the waste management system [29, 30]. Production volumes, however, may give a good indication of the emission of specific NP. Available data on production volumes differ greatly depending on the way of data collection. TiO_2_ NP and SiO_2_ NP are certainly the most relevant materials in terms of worldwide productions volumes (> 10,000 t/a in 2010), followed by CeO_2_ NP, FeO_*x*_ NP, AlO_*x*_ NP, and ZnO NP, and carbon nanotubes (CNT) (100–1000 t/a in 2010). The production volume of Ag NP was estimated with approximately 55 t/a worldwide in 2010 [31].

First attempts to estimate NP emissions during the life cycle indicated that most NP are emitted during use phase and after disposal, e.g. on landfills [30], while during production not more than 2% of the production volume is released [32]. Depending on the type and application of NP, they are either directly released into the environment, or indirectly via technical compartments and waste streams or enter in-use stock causing a delayed release [22, 30, 33–35]. The release pattern and masses depend on the NP type and its application. For instance, Sun et al. [22] studied emission patterns in the EU in 2014 of TiO_2_ NP, ZnO NP, Ag NP, and CNTs considering landfills, sediments, and soil as sink for NP. They found that TiO_2_ NP accumulate in sludge-treated soils followed by sediments and landfills (approx. 8400 t/a and 7600 t/a and 7000 t/a). The dominating emission pathway of TiO_2_ NP occurs via wastewater (85% of total TiO_2_ NP emissions) [30, 47]. For example, TiO_2_ NP are accumulated in sewage sludge during wastewater treatment, which is in many countries ultimately deployed onto soils. It was estimated that approximately 36% of TiO_2_ NP emissions occur via this pathway. A lower portion of the sewage sludge is deposited onto landfills directly or after incineration which equals approximately 30% of total emitted TiO_2_ NP. TiO_2_ NP emissions via wastewater effluent account for approximately 33% [22]. ZnO NP, which are mostly used in cosmetics, electronics, and medicine accumulate in sediments (1300 t/a), in natural and urban soil (300 t/a), as well as at landfills (200 t/a). The dominating emission pathway occurs, just as for TiO_2_ NP, via wastewater since both are used in cosmetics [47]. CNTs and Ag NP show different emission patterns. CNTs are predominantly emitted via production and use, and are directly deposited at landfills. Hence, approximately 90% of the CNT production is accumulated in landfills, approximately 10% in soils and < 1% in sediments and air [22]. Ag NP are emitted from production and use to both landfills and wastewater.

Global estimation of NP emissions indicates that landfills (approximately 63–91%) and soils (approximately 8–28%) receive the largest share followed by emissions into the aquatic environment and air (7 and 1.5%, respectively, of the production volumes) [30]. Such estimations allow to identify applications with potentially high environmental implication. A potential increase in outdoor applications of NP may elevate their mass flows directly into the aquatic and terrestrial environment [36]. For example, NP emission from façade paints, such as photocatalytic active compounds, e.g. TiO_2_ NP, has been demonstrated previously [19, 37, 38].

Besides emission of engineered NP, there are particulate emissions of anthropogenic NP which are not intentionally produced as NP. For instance, particulate emissions from traffic, such as palladium, were identified to be in nanoscale [39, 40]. NP release might also be intended due to their direct application in environmental compartments, for instance, for groundwater remediation such as iron-based NP [41–43] or when applying nano-pesticides directly to agricultural fields [44, 45]. Although some information on NP emission is available, it is of high importance to quantify their amounts and concentrations in the environment. Quantification of NP emissions into the aquatic environment has by now, however, been hampered by the lack of appropriate analytical techniques [5].

## Predicting and measuring nanoparticles in natural ecosystems

Computational modelling was suggested as a way forward estimating environmental concentrations because straight forward analytical methods were not available for detection of NP in the environment [46, 47]. Material flow models rely on life cycle information and production volumes, which were not necessarily available in sufficient detail, limiting their accuracy [48]. Very recently, more advanced models use probabilistic approaches [49] that consider dynamic input rates, in-use stocks as well as the continuous rise in production volumes [22]. These models allow predicting the time-dependent material flow of specific NP (e.g. TiO_2_ NP) in technical systems and environmental compartments. These models estimate NP concentrations in surface waters to be in the lower ng/L or µg/L range depending on the type of NP. For instance, mean NP concentrations in surface water were estimated for TiO_2_ with approximately 2.2 µg/L (Q_0.15_ 0.19 µg/L to Q_0.85_ 4.4 µg/L) and for Ag NP with 1.5 ng/L (Q_0.15_ 0.4 µg/L to Q_0.85_ 2.8 ng/L) for the EU in 2014 [22]. Although these models hardly consider NP-specific fate mechanisms (e.g. sedimentation [50]), first studies assessing the actual presence of NP in the aquatic environment [35, 36, 51, 52] are in consensus with modelling results [16]. For instance, analytical studies revealed TiO_2_ NP surface water concentrations between 3 ng/L and 1.6 µg/L, confirming the high variation of modelling results in a comparable concentration range. However, analytical limitations, namely the lack of specific and sensitive analytical methods in complex matrixes, did not yet allow to formally assess for the assumption that the increasing production and market volume of NP will ultimately lead to an increase in environmental concentrations.

Complementary analytical techniques have been used to determine and characterize metal-based NP in different environmental compartments [51, 53]. Concentration and size of metal-based NP such Au, Ag, Cu, TiO_2_, in surface water and soils have been, for example, determined by single particle inductively coupled plasma mass spectrometry (sp-ICP-MS) [53, 54] or fractionation techniques in combination with light scattering and elemental detection [55]. Structural information and information on particle size have been derived from electron microscopy as a complementary technique [51]. Among others, the NP surface chemistry including surface charge or functionalization controls NP fate. Therefore, surface characterization methods are important to understand NP fate processes [56].

For complex types of NP such as core shell structures a multi-element technique, e.g. sp-ICP-Time of Flight (ToF)-MS was developed [57] and has recently been successfully applied to determine engineered CeO_2_ NP in soil [58]. This technique is of high importance to differentiate between engineered NP and natural NP by detecting impurities in natural NP which are not present in engineered NP. Such analyses will help to validate model outputs on environmental NP concentrations.

In comparison to the analysis of inorganic NP in environmental compartments, organic NP analysis is still in its infancy. In fact, analytical techniques for organic NP have been developed, for example, for fullerenes and CNTs [52, 59] but they are hampered by insufficient selectivity with regard to the high environmental background concentrations of carbon. To improve the analysis of organic NP in complex media such surface water or soil, more efficient extraction methods are needed [60].

Recently, considerable progress has been made overcoming some of the analytical problems (natural particle counterparts, low concentrations, matrix interferences) [58, 61, 62], opening new possibilities that foster our understanding on both sources and fate of NP in the environment. However, future work should focus on the differentiation between engineered and natural NP, the detection of organic NP, and the characterization of the NP surfaces.

## Fate of nanoparticles in the environment

NP in the environment undergo ageing processes such as chemical transformation, aggregation, and disaggregation. The interplay between these processes and the NP transport determines the fate and ultimately the ecotoxicological potential of NP [63–65]. Since particle properties and environmental conditions control these ageing and transport processes [45, 65, 66], a direct transfer of data to even slightly deviating conditions is difficult and can even be misleading. Here, we analyse the current state of the art regarding NP fate and conclude if this knowledge is sufficient to allow for extrapolations from well-defined and controlled laboratory conditions to complex, real-world scenarios.

**Fig. 1:**
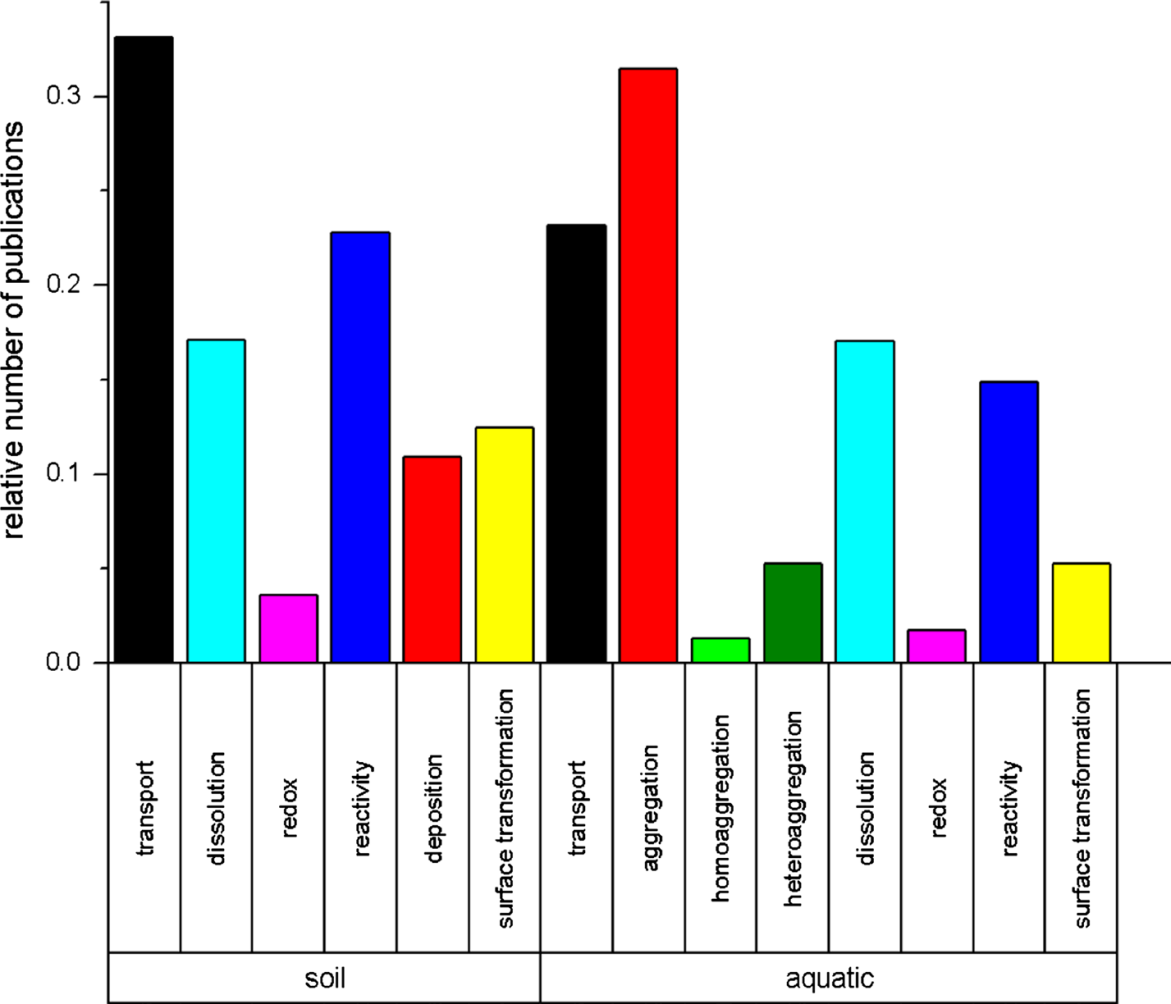
Relative number of publications compared to total number of publications found between 2007 and 2017 in soil and aquatic environments (search criteria: nano* & environ* & *system*, NOT effect* & NOT *tox*; process search criteria: transp*, agg*, homoagg*, heteroagg*, dissol*, redox*, surface* transfo*, reacti*, deposition*; system search criteria: soil*, aqua*; only web-of-science category *environmental science* considered)

**Fig. 2:**
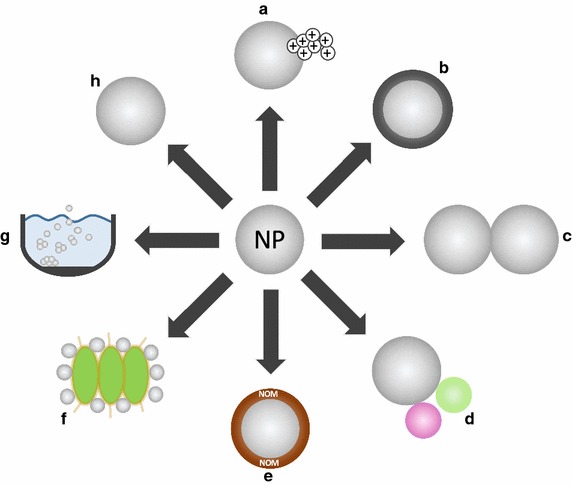
Interactions and fate of NP in the environment considering (a) dissolution, (b) sulfidation, (c) homo-aggregation, (d) hetero-aggregation, (e) coating with NOM, (f) NP adsorption on biological surfaces, (g) sedimentation/deposition, and (h) persistence

Alterations in chemical speciation, dissolution, degradation, as well as alteration of the surface properties by precipitation and ad- or desorption are important chemical transformation processes of NP, which have frequently been investigated both in aquatic and soil ecosystems (Fig. 1). A general remark in this context: functionalization of NP surfaces, which makes the particles’ properties more beneficial for industrial purposes [67, 68], will strongly control transformation processes in the environment [66, 69, 70]. A thorough characterization of NP surface chemistry (i.e. the formation or loss of coating [71]) over time seems therefore mandatory to understand NP fate [72, 73], but is still highly challenging. The present paper, however, specifically focuses on dissolution, passivation, aggregation, adsorption, sedimentation, and deposition as a selection of the most relevant processes under field conditions (see Fig. 2).

### Chemical transformation

Dissolution of NP (Fig. 2a) is driven directly by the particle chemistry. Dissolution of Ag NP, for instance, requires aerobic conditions. In such environments, an oxide layer (Ag_2_O) can be formed around the particle, which releases Ag^+^ [74]. In a range of studies, it was uncovered that Ag NP dissolution rates are triggered by particle-inherent factors including surface coating, size, shape, and state of aggregation, as well as environmental parameters such as pH, dissolved organic carbon, and temperature (see for a more detailed assessment of the mechanisms [11, 75–80]). Koser et al. [81] used equilibrium speciation calculations, based on a broad basis of literature data, to successfully predict Ag dissolution in several artificial media. This suggests that the scientific community was indeed capable of advancing the knowledge to a state that allows for reasonably accurate predictions and modelling.

A passivation process frequently occurring under various environmental conditions is the sulfidation of NP (Fig. 2b) that includes Ag NP, ZnO NP, and CuO NP [e.g. 79, 82, 83]. Sulfidation of Ag NP, for instance, can lead to the formation of core–shell Ag_0_–Ag_2_S structures or hollow Ag_2_S NP [84]. The mechanisms of sulfidation are given elsewhere [66, 84–86]. Sulfidation leads to nearly inert NP surfaces with consequence for their reactivity (Fig. 2) [84, 87] and thus toxicity [66], while sulfidized NP can still be toxic to microorganisms [88].

### Colloidal stability

Colloidal stability of NP is one of the key factors controlling their fate and effects [12, 89]. When released into the environment, NP interact with the variety of dissolved or particulate, inorganic or organic compounds influencing NP aggregation dynamics and thus colloidal stability [90]. Ultimately, exposure conditions are controlled by NP aggregation. By focusing on factors controlling homo-aggregation (interaction between the same NP) and hetero-aggregation (interaction between different NP or between NP and natural colloids such as montmorillonite, maghemite, kaolinite but also microorganisms, algae, and proteins [91, 92]), as well as disaggregation, we also discuss the processes determining NP fate.

Homo-aggregation of NP (Fig. 2c) is positively correlated with their concentration in the media. Since predicted environmental concentrations are rather low (in the pg/L to the low µg/L range for surface waters [16]), homo-aggregation is less likely due to the low probability for collisions. Nonetheless, this factor is relevant—but largely ignored—for laboratory-based ecotoxicological investigations that often use high NP concentrations compared to predicted environmental concentrations [76]. Under field conditions, the ionic strength of the surrounding medium seems more relevant as aggregation rates increase with ionic strength [e.g. 93, 94]. The aggregation dynamics are in most cases characterized by classical Derjaguin-Landau-Verwey-Overbeek (DLVO) theory [95, 96]. Furthermore, multivalent cations are more efficient than monovalent cations [97], whereas the efficiency within both groups of cations depends on their respective identity as judged by critical coagulation concentrations (CCCs): the aggregation of citrate-coated Ag NP was, for instance, more efficient for Ca^2+^ than Mg^2+^ [76], which may be explained by the higher ability of Ca^2+^ to form citrate complexes [98]. At the same time, the impact of cations and anions can be concentration dependent. Whereas high concentrations of Cl^−^ ions enhance the aggregation of Ag NP due to the bridging by AgCl [99], low concentrations may stabilize NP via the formation of negatively charged surface precipitates [76]. Similarly, soils and soil extracts modulate NP aggregation [100].

In addition to ionic strength, homo-aggregation is also influenced by a range of environmental variables. The surface charge of NP changes with pH, which is reflected by the isoelectric point (IEP; i.e. the pH at which NP do not carry a net charge). The IEP varies substantially among commercially available NP [101, 102], suggesting that even at the same pH the fate and thus the interaction of NP with organisms might vary substantially [103]. Furthermore, natural organic matter (NOM) can increase or reduce colloidal stability of NP as a function of its quality and quantity as well as the ionic strength of the medium [e.g. 104]: at low ionic strength, NOM stabilizes negatively charged NP through electrostatic and/or steric forces [76, 100]. Due to the formation of cation-NOM bridges among NP, NOM can enhance aggregation at high ionic strength [e.g. 97]. Positively charged NP may also increase aggregation in the presence of NOM as shown for the combination of negatively charged NOM and TiO_2_ NP due to charge screening [105]. Until now, the effects of several individual factors like surface coating of NP, ionic strength, and valence and type of cations on the fate of NP are characterized to reasonable degree. However, less is known on how the interplay between these individual factors affects the fate of NP under realistic environmental conditions.

In contrast to homo-aggregation, hetero-aggregation (Fig. 2d) is considered to be of higher environmental relevance given the several orders of magnitude higher concentration of natural colloids [106] relative to NP [16]. Quik et al. [107], for example, indicated that hetero-aggregation is the main mechanism removing CeO_2_ NP from the water phase through sedimentation. The aggregation kinetics of NP and natural colloids or other NP are particularly fast if they are differently charged [92, 108]. The presence of NOM, in contrast, reduces hetero-aggregation due to electrostatic and steric stabilization [109]. Besides electrostatic forces, bridging by polymers, hydrogen as well as chemical bonding were reported as mechanisms inducing hetero-aggregation. This process is, thus, highly complex and among others triggered by NP surface properties, their ageing status, interacting particulate phases, chemical composition of the surrounding environment, and the properties of natural inorganic, organic and biological colloids. However, only a few publications assessed aggregation in complex field-relevant media [e.g. 93, 100, 110], complicating any conclusion about the general relevance of the processes detailed above. Even less is known about the reversibility of NP aggregation in natural systems [93]. As disaggregation is mainly triggered by changing environmental conditions, experiments in simplified artificial systems are falling too short to properly address the dynamics of aggregation and disaggregation in real aquatic systems. This calls for experimental designs capable of simulating such fluctuating conditions. Furthermore, low and environmentally relevant NP concentrations should be used in future studies to avoid potentially confounding implications of NP homo-aggregation, which is, as outlined above, less likely under currently predicted environmental concentrations of NP compared to hetero-aggregation.

### Transport in porous media

Most of the published data on NP mobility in porous media was generated using water saturated artificial columns frequently made of quartz sand [e.g. 111] while only a few involved natural soil [e.g. 92]. Similar to aquatic ecosystems, ionic strength triggers NP’ fate: NP retention and deposition in porous media (Fig. 2) is positively related to the ionic strength of the pore water [112], likely driven by quick NP aggregation [e.g. 111]. At high levels of ionic strength, retention rates can further increase due to the “ripening effect” (i.e., increasing attraction forces between NP in the liquid phase and NP already deposited onto soil [113]). Hetero-aggregation of Ag NP with soil colloids, in contrast, can even enhance NP mobility by the size-exclusion effect [92, 112]. Furthermore, electrostatic and steric repulsion forces induced by NOM often lead to higher mobility [e.g. 112, 114], while a decreasing pH has the opposite effect [e.g. 111].

In contrast to water saturated systems, unsaturated porous media are less frequently but increasingly assessed (see for instance, [115]). Relative to water saturated porous media, unsaturated systems have often a higher retention potential [e.g. 116], while Fang et al. [117] found only marginal differences regarding the retention of TiO_2_ NP between saturated and unsaturated porous media. This pattern may be explained by the non-equilibrium sorption to solid–water interface and equilibrium sorption to air-water interface [118]. Moreover, the water film straining during drying could increase NP retention [116] and is thus of potentially high relevance in unsaturated porous media. The impact of ionic strength, type of cations, NOM but also pH on NP fate is comparable among aquatic systems and porous media irrespective of whether the latter is saturated or unsaturated. Therefore, we refer to the subchapters above for a more detailed description.

Driven by the few number of studies [e.g. 100] and the partly contradictory outcomes particularly for unsaturated porous media, a reliable prediction of NP fate in soil ecosystems seems difficult. Thus, a more systematic approach is urgently needed uncovering the role of soil properties on NP fate. One important step would be to assess NP fate in natural soils instead of porous media made of artificial substrate such as quartz sand. Although there are certainly challenges from the analytical side, i.e. inorganic NP might occur at high levels in natural soils and form a substantial background contamination, which can be overcome with the help of modelling, those data are likely of higher field relevance. In natural soils, for instance, microorganisms, inorganic and organic particles might form complex bio-geochemical interfaces that interact with NP and as a consequence will influence their fate [119] and toxicity. Those insights can, however, barely be inferred from experiments using highly artificial substrates. On the way to indeed using natural soils, it may however, be feasible to employ more complex artificial soils containing among others also natural organic matter.

In summary, predominantly qualitative information on particle fate in aquatic and porous media is available and key factors which control fate processes have been identified. Particle surface properties and NP concentration are most relevant and need to be characterized carefully—also throughout the experiments. Environmental key factors controlling NP fate processes are ionic strength, type and charge of ions, pH, type and concentration of NOM in all environmental compartments, while the degree of water saturation is an additional factor for porous media. Quantitative systematic data are rare [12, 45, 50, 64] but fundamental for process understanding and ultimately for the development of reliable and predictive fate models. Future studies should, thus, qualitatively and quantitatively assess NP fate under environmentally relevant conditions, reflecting amongst others not only realistic NP concentrations, environmental conditions but also reaction and residence times. These data will certainly support the recent developments in NP fate modelling, namely simulations of individual fate processes and fate predictions in rivers and porous media [64, 120–123]. As their validation is largely lacking, this aspect should be addressed in future work.

## Effects of nanoparticles and their mechanisms of toxicity

**Fig. 3:**
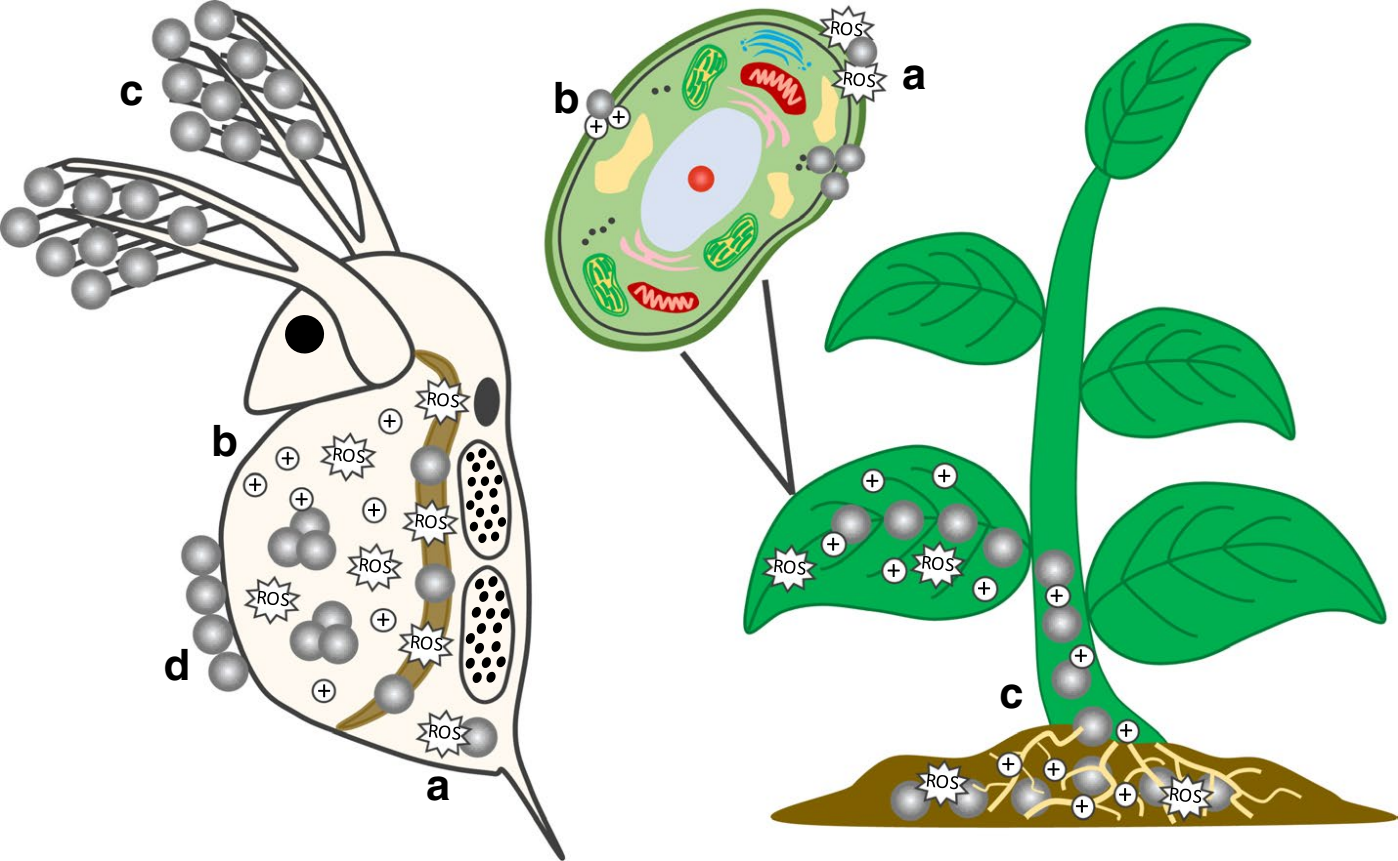
Potential ecotoxicity of NP in aquatic and terrestrial regimes, illustrating locally acting mechanisms as **a** formation of ROS, **b** ion release, **c** internalisation, and **d** biological surface coating

Roughly a decade ago and thus with the initiation of the research field “nano-ecotoxicology”, Moore [124] as well as Hund-Rinke and Simon [125] suggested that NP have the potential to cause harmful effects in biota by the formation of reactive oxygen species (ROS) that could affect biological structures (Fig. 3a). Moore [124] also pointed to the potential of NP to function as carriers for other pollutants (Fig. 2d)—an assumption that will be addressed in more detail in the next chapter. While it is evident from the literature that oxidative stress can indeed be a driver for many NP-induced effects [126], the last decade of research showed that NP have the ability to act via multiple pathways of which the induction of oxidative stress is one. In the following, we briefly highlight the current state of the art with regard to central aspects supposedly driving the ecotoxicity of NP.

No mechanism of toxicity can be considered as generic for all NP [126]. Oxidative stress is, however, a frequently reported phenomenon [127, 128]. Just to name a few other relevant mechanisms, physiological implications that can go as far as reproductive failure by modifying hormones or hatching enzymes were reported [129, 130]. Those effects indicate implications in population development and suggest the potential for transgenerational effects [131, 132]. In addition, algae [133] and aquatic plants [134] were altered in their photosynthetic pigment composition and showed effects in photosystem II, while we refer to Thwala et al. [135] for a more detailed review. Similarly, several recent reviews have covered NP accumulation in terrestrial plants which can cause biochemical and physiological changes [136–138]: Cao et al. for instance, documented impacts on carbon fixation as well as water use efficiency during photosynthesis in response to CeO_2_ NP exposure [139]. The latter may have indirect effects on soil organisms via implications in soil moisture. Besides this massive diversity regarding the mechanisms of toxicity among NP, species and ecosystems, some more general questions attracted attention among researchers, namely the relevance of ions released (dissolved) from NP for NP-induced effects.

## Effects of nanoparticles on individuals and populations

### Effects of ion-releasing NP

Certain NP are prone to dissolution, i.e. the release of ions from the NP surface, during their entire (aquatic) life cycle (Figs. 2, 3) [140]. In such cases, researchers were interested in uncovering the relevance but also the mechanism of toxicity induced by those ions released from NP. Against this background, Ag NP have frequently been assessed, suggesting that Ag ions released from these NP explained a large proportion of the observed toxicity for various test organisms [e.g. 141–143] and soil microbial communities [144]. Additionally, the mechanisms of toxicity, for example, for snails [143] and periphyton [141], were largely comparable between Ag ions and Ag NP. These observations contradict other findings, highlighting more severe implications by Ag NP than what could be explained exclusively by the Ag ions measured [145]. Differences in gene expression and transcriptomic profile point towards distinct mechanisms of toxicity in aquatic [146, 147] and terrestrial organisms [148]. Nonetheless and in line with the extensive literature review by Völker et al. [149] it may be suggested that Ag NP and Ag ions share common mechanisms of action. This conclusion also implies that the Ag NP-induced toxicity, which largely depends on the particle surface properties, diameter, and exposure time [150], can mainly be explained by the quantity of released ions [sensu 151, 152]. Ag NP are, moreover, sulfidized (see above, Fig. 2b) in wastewater treatment plants and trapped in sewage sludge. As a consequence of the usage of sewage sludge as fertilizer for crop production in various countries, soil organisms are directly exposed to Ag_2_S NP, Ag NP, and Ag^+^ ions. The form of Ag directly influences the location and speciation (metallic, ionic, thiol, NP) in which Ag is stored in wheat roots [153]. In cucumber and wheat, Ag_2_S NP remained in their NP form and were translocated from the roots to leaf tissue, reducing plant growth and activating plant defence mechanisms [154]. Internalization (Fig. 3c) or physical adherence, such as biological surface coating (inhibiting, e.g. photosynthesis, nutrient uptake or movement) are also considered as possible mechanisms of toxicity for “inert” NP, i.e. those not releasing toxic ions (Fig. 3d) [155, 156].

CuO NP exposure resulted in a different pattern relative to Cu ions. This was shown for protein regulation and gene expression in marine mussels [157] and zebrafish gills [146]. Similarly, Mortimer et al. [158] indicated CuO particle-related effects in the membranes’ fatty acid composition of protozoa pointing towards distinct mechanisms of toxicity induced by Cu ions relative to CuO NP. Also in wheat grown on sand, CuO NP reduced root length and simultaneously increased root hair length with no effect on shoot growth, while Cu ions reduced both root and shoot growth [159]. A recent study by Pradhan et al. [160], however, suggests that aquatic fungi from metal contaminated ecosystems have a higher tolerance towards CuO NP driven by elevated enzymatic activity. This observation may indicate a specific adaptation of fungi towards Cu ions, supporting fungi to withstand CuO NP stress, which might indicate a common mechanism of toxicity. On the other hand, a more generic defence mechanism might have been strengthened by the long-term adaptation towards metal stress in general, which resulted in an evolution of co-tolerance towards CuO NP.

Similar to CuO NP, the mechanism of toxicity induced by ZnO NP seems to deviate from its ionic counterpart. Fernandez-Cruz et al. [161], for instance, suggested that cytotoxic effects in fish cells are mainly particle-related, which is supported by studies uncovering difference in gene expression [162, 163] and thus different mechanisms of toxicity [164]. Recent evidence with soil nematodes (*Caenorhabditis elegans*) indicates a higher ecotoxicological potential of ZnO NP relative to Zn^2+^ ions [165].

It is hence not possible to draw a generic conclusion regarding the relevance of toxic metal ions released from NP relative to the effects induced by the NP themselves. This relationship is rather driven by the NP identity and coating, the biological system, and the environmental conditions (e.g. complexation with NOM) under which the studies are performed. For instance, gene expression profiling in enchytraeids (*Enchytraeus crypticus*) suggested deviating mechanisms of toxicity for different Ag NP products [166]. Also the uptake and toxicity of Ag NP in earthworms are triggered by the particle coating. Makama et al. [167] reported size-dependent effects only for PVP-coated Ag NP. The uptake of Ti through the roots into basil was highest when exposed to TiO_2_ NP with hydrophobic relative to hydrophilic coatings, causing alterations in the content of several essential elements and sugar [168]. A long-term study with two agricultural soils and different plant species looked at the effects of ZnO NP [169]. In this study, acidic soils stimulated Zn accumulation, ROS production, and photosynthetic pigments of beans relative to calcareous systems, while the opposite pattern was observed in tomatoes [169].

### Effects of NP not releasing toxic ions

For NP that are only marginally or not releasing toxic metal ions during the aquatic life cycle, “biological surface coating” (i.e. the attachment or adsorption of NP to the organisms’ outer surface, Fig. 3d) is suggested as potential toxicity trigger [170]. The acute toxicity of TiO_2_ NP and Fe_3_O_4_ NP in daphnids, for instance, was attributed to a physical inhibition of moulting, ultimately inducing death [155, 156]. At the same time, biological surface coating could alter daphnids’ swimming behaviour with potential chronic effects [171]. These effects are usually more pronounced at smaller initial particle sizes, while evidence suggest that the surface area is the driving force for toxicity [7, 172, 173].

## Nanoparticle-induced effects over multiple generations

As highlighted earlier, aquatic invertebrates have shown an increase in sensitivity in filial generations as a consequence of the exposure of the parental [131] or subsequent generations [132] towards TiO_2_ NP. Similarly, at Au NP concentrations inducing only negligible mortality in the parental generation of the soil organism *C. elegans*, the reproduction of subsequent generations (F1–F4) was substantially impaired [174]. In a full lifespan test, CuO NP caused more severe effects compared to a standard test duration [175]. Also, plants exposed to CeO_2_ NP in their parental generation showed response in the filial generation with implications in the grains’ nutrient composition, growth, and physiology [176]. Despite the current lack of mechanistic understanding regarding the underlying processes, these insights support the idea of long-term effects in natural populations of aquatic and terrestrial organisms. Importantly, such effects are not covered by most standardized test systems. More systematic research is needed addressing the mechanistic basis of these phenomena as well the evolutionary potential of populations and communities to adapt to these emerging stressors.

## Interactions of nanoparticles in a complex environment

### Impact of natural organic material on NP‑induced effects

In the environment, NP will interact with their abiotic surrounding, which influences their fate and ecotoxicological potential [81, 177]. The relevance of natural organic molecules attaching to NP (Fig. 2e) has recently been reviewed elsewhere [178], which is why we give only a few examples here. Dissolved organic matter (DOM) coats NP and thereby stabilizes particle size [179] due to steric or electrostatic repulsion [180]. These processes are more effective with increasing hydrophobicity or aromaticity of the DOM, ultimately reducing their ecotoxicological potential—likely by reducing the availability of reactive surfaces [181]. In situations where artificial (e.g. polyvinylpyrrolidone, gum arabic or citrate) or natural OM coats Ag NP, the release of potentially toxic ions into the surrounding environment [66, 182] or the NP bioavailability [183] is reduced. On the contrary, humic acid elevated the release of ions from Pb NP [184]. Bicho et al. [185] reported that soil and soil–water extracts could elevate effects of NP (here Europium polyoxometalates encapsulated in SiO_2_ NP) relative to their absence.

Thus, it becomes apparent that soil properties influence the toxicity of NP to soil organisms. Especially the organic matter content, soil texture, ionic composition, and pH affect the fate and bioavailability of NP [112, 186, 187], which leads to differences in toxicity. With a decrease in organic carbon in standard Lufa soils, the toxic effects of PVP-coated Ag NP to *E. crypticus* increased [188]. Less toxicity of AgNP was also observed for an entire test battery of dicotyledonous and monocotyledonous plants, Collembola and earthworms in soil with higher silt than sand content [189]. With a rise in soil pH, ZnO NP effects on *Folsomia candida* reproduction decreased [190].

When considering time as a factor, ageing of NP in presence of DOM for 48 h reduced or did not influence the toxicity of Ag and ZnO NP, respectively [191]. In contrast, the ageing of TiO_2_ NP in the presence of DOM for 1 and 3 days slightly increased toxicity for daphnids, while ageing for longer time periods (e.g. 6 days) reduced NP-induced effects substantially as NP agglomerates exceeded the size range retained by daphnids’ filter apparatus [192]. Similarly, Collembola (*F. candida*) showed more severe effects in their reproductive output with increasing ageing duration of Ag NP in spiked sewage sludge [193]. It is suspected that this is due to the continuous release of Ag ions from Ag NP.

Algal cells fed to invertebrates may enhance the uptake and toxicity of NP [194]. The adsorption of NP to algal cell surfaces (Fig. 2f) could accelerate their sedimentation (Fig. 2g), which forces pelagic consumers to invest more energy in collecting their food near the sediment [195]. Bundschuh et al. [196], in contrast, uncovered an increase in *Daphnia* growth and partly reproduction after allowing an interaction between algae and TiO_2_ NP for 1–3 days. Moreover, NP ingested together with the food could negatively influence digestion by clogging the gut with negative consequences on life history strategies of primary consumers in autotrophic food webs [195]. Similar patterns have been observed for detritus-based food webs [197]. Altogether, these observations suggest highly complex interactions between organic material (irrespective whether of particulate or dissolved nature), NP and biota, but are also pointing to a lack of mechanistic understanding.

### NP-triggered alterations in the ecotoxicity of co-occurring contaminants

Aquatic and terrestrial ecosystems are also commonly exposed to mixtures of chemical stressors, which raised concerns about the potential of NP to act as carriers for organic and inorganic chemical stressors of anthropogenic origin [198]. Schwab et al. [199] reported, for instance, an elevated herbicide (diuron)-induced toxicity applied at environmentally relevant concentrations in the presence of carbon-based NP. Similarly, the acute toxicity of the insecticide bifenthrin was increased in the presence of fullerene NP, while its chronic effects were not affected [200]. In line with these observations, a recent review suggested that carbon-based NP could also act as Trojan horse for metal ions. The potential of carbon-based NP to act as “Trojan horse” for other contaminants strongly depends on the characteristics of the surrounding environment (see for a more detailed discussion [201]). This conclusion is challenged by Sanchis et al. [202], reporting for most combinations of carbon-based NP and organic co-contaminants antagonistic effects for daphnids and bacteria.

Also, the combined exposure of metallic NP with other chemical stressors delivered contradictory results. The presence of TiO_2_ NP reduced the uptake of phenols [203] and polycyclic aromatic hydrocarbons [204], which, however, did not necessarily reduce the combined toxic effects of those organic chemicals and TiO_2_ NP, suggesting a significant contribution of the NP to the biological responses. On the contrary, the accumulation of perfluorooctanesulfonate in fish was facilitated in the presence of TiO_2_ NP. This pattern was particularly pronounced at the bottom of the experimental systems as a consequence of the adsorption of perfluorooctanesulfonate onto TiO_2_ NP surfaces and the subsequent aggregation and deposition of perfluorooctanesulfonate-loaded NP [205]. Similarly, TiO_2_ NP increase the uptake of metal ions [206] from the water phase and at high concentrations of OM also from sediments [207], in biota ultimately elevating biological responses.

Other publications indicate the opposite pattern, namely a reduction in metal ion-induced effects in the presence of nTiO_2_ or Al_2_O_3_ NP in algae [208, 209] or mussels [210], with an even more pronounced reduction in the presence of DOM [211]. Follow-up studies suggested that the direction and magnitude of effects caused by a combined exposure of TiO_2_ NP and metal ions are triggered by the charge of the most toxic metal ion [212]. Although the underlying mechanisms are not well understood yet [213] and the NP concentrations used in those experiments often exceed environmentally relevant concentrations by at least one order of magnitude, interactions of NP with co-contaminants will occur most likely under field conditions. The relevance of such interactions in both direction and magnitude for effects caused by co-contaminants in wildlife remains to be resolved.

## NP effects in communities and consequences for trophic interaction

McKee and Filser [214] reviewed interactions of metal-based NP in soils and pointed out the relevance of species interactions for fate and bioavailability of these NP, next to abiotic parameters. They stated that particularly biotic interactions might explain the negative consequences of NP on ecosystem processes such as carbon dioxide emission, nitrogen or phosphorous fluxes [215] that could not be detected from short-term, single species tests [214]. Button et al. [216], for instance, did not detect any negative effects on microbial community structure and function in wetland systems when exposed for 28 days to ionic Ag, citrate- and PVP-coated Ag NP (at 100 µg/L). These microbial communities, however, developed an Ag resistance, indicating an existing potential to adapt to such emerging stressors by elevating the production of extracellular polymers [217], while the costs of this adaptation remain unclear. Evidence is increasing that nitrifiers are among the most sensitive microorganisms towards Ag NP [218–220], even when the NP are sulfidized [88]. Nitrite production rate was also reduced by about 30% in the presence of 200 mg magnetite (Fe_3_O_4_) NP/L [219], a concentration considered as rather low in soils remediated from organic contamination [41]. This relatively low-effect threshold (given that iron oxides are mostly being considered harmless to beneficial) is supported by a range of further studies reporting sometimes surprisingly strong negative effects on microorganisms exposed to iron oxide NP [214, 221, 222].

When actively spraying Ag NP as growth promoter on several plant species, root and shoot mass of cowpea and *Brassica* increased, while only for the latter in a dose-dependent manner [218]. For wheat, in turn, shoot mass remained stable and root mass decreased with increasing Ag NP exposure [218]. These authors also reported increased bacterial counts and higher abundances of P-solubilizing bacteria in pure soil (without plants) following Ag NP exposure while at higher Ag NP concentration the abundance of N-fixing bacteria decreased. In the rhizosphere of plants treated with Ag NP, however, the microbial responses varied with plant species and thus deferred from the pure soil [218]. An experiment with Fe_3_O_4_ NP and *Zea mays* documented a reduction in the diversity of arbuscular mycorrhizal fungi accompanied by a decreased catalase activity, plant biomass, phosphorous content, carbon transfer to the fungi, and impaired root colonization and phosphatase activity [221]. These examples emphasize that the interactions between soil microorganisms, plants, and NP are highly complex, warranting further research. Addressing the underlying processes will ultimately support a well-informed decision making about potential environmental risks of NP when applied to agricultural fields.

Recent studies highlight the potential for indirect effects of NP on invertebrate species via microorganisms. Antisari et al. [223] reported for Co NP a reduction in soil microbial biomass and changes in its community composition, which may partly explain alterations in earthworms’ fatty acid composition. The uptake of TiO_2_ NP via contaminated food seems to be of high relevance for terrestrial [224, 225] and aquatic invertebrates [226]. This direct uptake pathway increases NP exposure and might influence the food quality through impacts on food-associated microorganisms.

In aquatic systems, structural [227, 228] and functional [141, 229] changes, such as photosynthetic efficiency and leaf decomposition were reported, although often at rather high concentrations. These examples suggest that NP can indeed affect species but also species interactions [230] at various trophic levels. At the same time, neither the mechanisms driving these changes nor the consequences for the wider food web or whole ecosystems have yet even been addressed.

## Conclusion

Over the past decade, our understanding of sources, fate, and effects of NP in the environment has made significant progress. Besides the call to consider environmental relevant concentrations of NP as well as to monitor the fate of NP during biological testing, there are multiple open questions that need further consideration. A more systematic approach is urgently needed uncovering the role of soil properties (including saturated and unsaturated systems) for NP fate and thus the risk of groundwater contamination by NP. We have, for instance, learned that ions released from NP can in some situations fully explain the effects observed in organisms. It is currently, however, not possible to properly describe under which conditions this simplified assumption should be rejected and other mechanisms need to be considered. The impact of coating—either as intended functionalization or based on natural processes—on the fate and effects of NP is currently underrepresented in literature (but see, [231]). As those coatings are likely of high field relevance, we strongly recommend their inclusion in future projects. Although most studies highlight effects at relatively high NP concentrations, more recent approaches document sublethal implications at field-relevant levels particularly over multiple generations (as reviewed in [177]). Thus, the impact of NP under current and future exposure scenarios (including co-exposure to other stressors) on communities, ecosystems, ecosystem functions as well as interactions across ecosystem boundaries deserves special attention. Particularly for sparingly soluble or insoluble NP that may accumulate in certain environmental compartments (e.g. sediments) over time, investigations covering multiple years of (repeated) exposure and assessment are suggested to properly assess their potential long-term implications in aquatic and terrestrial ecosystems. This aspect directly links to the acknowledgement of NP-induced alterations in horizontal and vertical trophic interactions with food webs.
